# Stereocomplex-Reinforced PEGylated Polylactide Micelle for Optimized Drug Delivery

**DOI:** 10.3390/polym8040165

**Published:** 2016-04-22

**Authors:** Chunsheng Feng, Meihua Piao, Di Li

**Affiliations:** 1 Department of Anesthesiology, The First Hospital of Jilin University, Changchun 130021, China; csfeng@jlu.edu.cn; 2 Department of Chemistry, Northeast Normal University, Changchun 130024, China

**Keywords:** controlled drug delivery, malignancy therapy, polylactide, self-assembly, stereocomplex interaction

## Abstract

The instability of PEGylated polylactide micelles is a challenge for drug delivery. Stereocomplex interaction between racemic polylactide chains with different configurations provides an effective strategy to enhance the stability of micelles as the nanocarriers of drugs. In this work, a stereocomplex micelle (SCM) self-assembled from the amphiphilic triblock copolymers comprising poly(ethylene glycol) (PEG), and dextrorotatory and levorotatory polylactides (PDLA and PLLA) was applied for efficient drug delivery. The spherical SCM showed the smallest scale and the lowest critical micelle concentration (CMC) than the micelles with single components attributed to the stereocomplex interaction between PDLA and PLLA. 10-Hydroxycamptothecin (HCPT) as a model antitumor drug was loaded into micelles. Compared with the loading micelles from individual PDLA and PLLA, the HCPT-loaded SCM exhibited the highest drug loading efficiency (DLE) and the slowest drug release in phosphate-buffered saline (PBS) at pH 7.4, indicating its enhanced stability in circulation. More fascinatingly, the laden SCM was demonstrated to have the highest cellular uptake of HCPT and suppress malignant cells most effectively in comparison to the HCPT-loaded micelles from single copolymer. In summary, the stereocomplex-enhanced PLA–PEG–PLA micelle may be promising for optimized drug delivery in the clinic.

## 1. Introduction

Polylactide (PLA) derived from lactic acid is known as one kind of biodegradable and biocompatible aliphatic polyester and is classified as a grade generally recognized as safe (GRAS) by US Food and Drug Administration (FDA) [1]. The easy clearance and good security make PLA a great potential candidate for biomedical applications, including medical devices [2], tissue engineering [3,4], drug delivery [5,6], and so on.

For successful use of PLA in drug delivery, hydrophobicity is one of the serious challenges. The hydrophobicity of PLA is not only difficult to be dispersed in water but also easy to be uptaken by a mononuclear phagocyte system (MPS), resulting in the limitation in circulation [7]. One of the most effective approaches to address this problem is to introduce hydrophilic blocks, such as polyzwitterions and poly(ethylene glycol) (PEG) into PLA, synthesizing amphiphilic PLA-containing copolymers [7]. The PLA-based copolymers can self-assemble into micellar nanoparticles in aqueous condition induced by microphase separation [8,9]. The hydrophilic coronas ensure the water-solubility, anti-protein adsorption, and long circulation time, and the hydrophobic PLA regions serve as a reservoir of hydrophobic drugs. Along the way, the PEGylated poly(d,l-lactide) (PDLLA) micelle has been employed as the nanocarrier of paclitaxel (PTX) and clinically used in Korea (Trade name: Genexol^®^-PM) [10].

With the development of PLA-containing micellar drug delivery systems, their poor stability impeded their extensive application in the clinic. Chemical crosslinking either core or shell segments is a common strategy to improve the stability of polymeric micelles [11,12]. However, the preparation process may introduce some additional chemicals and then affect the biodegradability and biocompatibility of micelles [11]. More fascinatingly, various noncovalent interactions, such as hydrophobic, electrostatic, host-guest, and stereocomplex interactions, have been introduced as “green” crosslinking points to enhance the stability of polymeric micelles [13].

Among them, the formations of physical crosslinkers in polymeric micelles through stereocomplex formation have gained much more attention for their satisfactory characteristics [14]. Due to the formation of stereocomplexation, the PLA-originated micelles exhibit both strong thermodynamic and kinetic stability, and are less prone to aggregation during the lyophilization process [15,16]. Moreover, the release of loaded agents from the stereocomplex PLA micelles is much slower than the corresponding single component micelles without stereocomplexation [17,18]. Although the stereocomplex PLA micelles have been demonstrated to exhibit attractive advantages as aforementioned, the mechanism of PLA stereocomplex interaction in the core of micelles and the actual efficacy in drug delivery have rarely been systematically revealed.

**Scheme 1:**
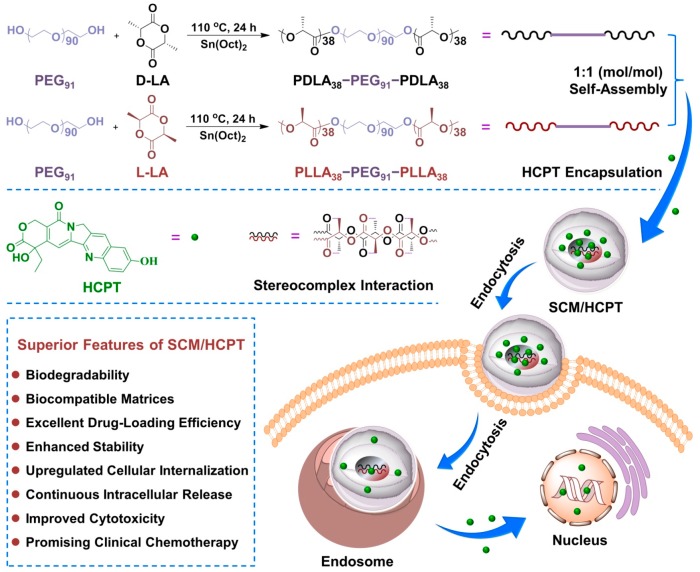
Schematic illustration for preparation, endocytosis, and intracellular drug release of SCM/HCPT.

In this work, two enantiomeric PLA–PEG–PLA triblock copolymers, that is, poly(d-lactide)-*block*-poly(ethylene glycol)-*block*-poly(d-lactide) (PDLA–PEG–PDLA) and poly(l-lactide)-*block*-poly(ethylene glycol)-*block*-poly(l-lactide) (PLLA–PEG–PLLA), were precisely synthesized (Scheme 1). The self-assembly and microcrystalline behaviors of PDLA–PEG–PDLA (PDM), PLLA–PEG–PLLA (PLM), and stereocomplex micelle (SCM) of the above two equimolar copolymers were systematically revealed. Subsequently, 10-hydroxycamptothecin (HCPT), as a model antitumor drug, was loaded into PDM, PLM, and SCM by a nanoprecipitation technique. The loading micelles were marked as PDM/HCPT, PLM/HCPT, and SCM/HCPT, respectively. In addition, the loading SCM micelle exhibited a sustained drug release profile in normal physiological condition, and efficient internalization and proliferation inhibition toward MCF-7 cells. The results indicated that SCM with upregulated stability and intracellular drug release exhibited great potential in controlled drug delivery.

## 2. Materials and Methods

### 2.1. Materials

PEG (number-average molecular weight (*M*_n_) = 4000 g·mol^−1^), stannous (II) 2-ethylhexanoate (Sn(Oct)_2_), 4′,6-diamidino-2-phenylindole (DAPI), Alexa Fluor 488 phalloidin (Alexa 488), and 3-(4,5-dimethylthiazol-2-y1)-2,5-diphenyltetrazolium bromide (MTT) were purchased from Sigma-Aldrich (Shanghai, China). d-LA and l-LA were gained from Changchun SinoBiomaterials Co., Ltd. (Changchun, China) and recrystallized from ethyl acetate under argon (Ar) atmosphere before use. HCPT was purchased from Huangshi Li Shizhen Pharmaceutical Group (Huangshi, China). Toluene was dried and distilled with sodium/benzophenone under a nitrogen atmosphere before use. Clear 96-well tissue culture polystyrene (TCP) plates were purchased from Corning Costar Co. (Cambridge, MA, USA). The deionized water used in this study was prepared through a Milli-Q water purification equipment (Millipore Co., Billerica, MA, USA).

### 2.2. Syntheses of PDLA–PEG–PDLA and PLLA–PEG–PLLA Triblock Copolymers

The triblock copolymers of PDLA–PEG–PDLA and PLLA–PEG–PLLA were synthesized by the ring-opening polymerization (ROP) of d-LA and l-LA, respectively, initiated by PEG and catalyzed by Sn(Oct)_2_ according to the previously reported proposal [19,20]. In brief, 10.0 g of PEG containing 5.0 mM hydroxyl group (–OH) dissolved in toluene was azeotropically distilled at 120 °C to remove the micro-amount of water. Subsequently, DA or LA (14.4 g, 0.1 mol), Sn(Oct)_2_ (40.5 mg, 0.1 mmol), and 150.0 mL of toluene were added to the above system. The reaction was performed at 100 °C for 24 h. The reaction solution was precipitated into over excess diethyl ether, and redissolved in chloroform and reprecipitated in diethyl ether. Finally, the obtained PDLA–PEG–PDLA and PLLA–PEG–PLLA triblock copolymers were dried under vacuum to a constant weight at room temperature. The yield was more than 90%.

### 2.3. Micelle Preparations

For the experiments, 0.1 g of PDLA–PEG–PDLA or PLLA–PEG–PLLA copolymer, or the above two equimolar copolymers was dissolved in 40.0 mL of tetrahydrofuran (THF) individually. After stirring for 5 h, 25.0 mL of deionized water was dropwise added into the above copolymer solution at a rate of 0.1 mL·min^−1^. After stirring overnight, THF was removed from the solution by dialysis (molecular weight cut-off (MWCO) = 3500 Da) against deionized water for 24 h to obtain the micellar solution.

### 2.4. HCPT Encapsulations

The drug-loaded micelles were prepared through a proposal of nanoprecipitation. Typically, 0.5 g of copolymer was first dissolved in 40.0 mL of THF, and then 75.0 mg of HCPT was added with vigorous stirring. Subsequently, 30.0 mL of deionized water was dropwise added at a rate of 0.1 mL·min^−1^. After stirring overnight, THF and free HCPT were removed by dialysis (MWCO = 3500 Da) for 24 h, and then the obtained loading micelle solution was lyophilized to obtain the solid drug-loaded micelle.

The drug loading content (DLC) and drug loading efficiency (DLE) of HCPT were determined by absorption at the wavelength of 365 nm (HCPT) on a Shimadzu UV2401 ultraviolet-visible (UV–Vis) spectrophotometer (Shimadzu, Kyoto, Japan) and were calculated by the following Equations (1) and (2), respectively:

(1) $$\mathrm{DLC}\;(\mathrm{wt}\%)=\frac{\text{Weight of drug in micelle}}{\text{Weight of drug-loaded micelle}}\times 100\%,$$

(2) $$\mathrm{DLE}\;(\mathrm{wt}\%)=\frac{\text{Weight of drug in micelle}}{\text{Total weight of feeding drug}}\times 100\%.$$

### 2.5. Measurements

Proton nuclear magnetic resonance (^1^H NMR) spectra were recorded on a Bruker AV 400 NMR spectrometer (Bruker BioSpin, Rheinstetten, Germany) with deuterated chloroform (CDCl_3_) as a solvent. Gel permeation chromatography (GPC) analyses were performed using a series of linear Styragel columns (HT3 and HT4) and a Waters 515 HPLC pump with OPTILAB DSP Interferometric Refractometer (Wyatt Technology, Wellensieck, Minden, Germany) as a detector. The eluent was chloroform at a flow rate of 1.0 mL·min^−1^, 40 °C. Monodisperse polystyrene standards were used to generate the calibration curve and were purchased from Waters Co. Ltd. (Milford, MA, USA) with a molecular weight range of 1790–4.0 × 10^5^ g·mol^−1^.

The morphologies and sizes of blank micelles were investigated by transmission electron microscopy (TEM), which were carried out on a JEOL JEW-1011 instrument (Manufacturer, Tokyo, Japan) at an accelerating voltage of 100 kV. In addition, 10.0 μL of micellar solution (0.1 mg·mL^−1^) was dripped on copper grid and then dried at room temperature in the air. The hydrodynamic diameters (*D*_h_s) of blank and laden micelles were measured by dynamic light scattering (DLS) at 25 °C on a WyattQELS apparatus (Wyatt Technology Corp., Santa Barbara, CA, USA). The critical micelle concentrations (CMCs) of micelles in aqueous medium were estimated by fluorescence spectroscopy using pyrene as a probe on a Photon Technology International (PTI) Fluorescence Master System with the software of Felix 4.1.0 (PTI, Lawrenceville, NJ, USA).

The micellar solutions were lyophilized to obtain the solid PDM, PLM, and SCM for the detections of Fourier-transform infrared (FT-IR) spectra, differential scanning calorimetry (DSC), and wide-angle X-ray diffraction (WAXD).

Fourier-transform infrared (FT-IR) spectra were recorded on a Bio-Rad Win-IR instrument (Bio-Rad Laboratories Inc., Cambridge, MA, USA) using potassium bromide method.

The thermal properties of lyophilized micelles were measured by differential scanning calorimetry (DSC). The DSC measurements were conducted under nitrogen using a heat/cool/heat cycle at a heating rate of 5 °C·min^−1^ on a TA Instruments DSC Q100 (TA Instruments, Wilmington, DE, USA) with aluminum pan.

Wide-angle X-ray diffraction (WAXD) measurements were performed on a Rigaku D/max-A X-ray diffractometer (Tokyo, Japan) with a Ni-filtered Cu Kα radiation (λ = 0.154 nm) at room temperature. The scan was performed from 5 to 40 °C at a rate of 5 °C·min^−1^. The selected voltage and current were 40 kV and 200 mA, respectively.

### 2.6. In Vitro HCPT Release

The release profiles of HCPT-loaded micelles were assessed in 0.01 M phosphate-buffered saline (PBS) at pH 7.4, a mimicking normal physiological condition. Briefly, 1.0 mg of PDM/HCPT, PLM/HCPT, or SCM/HCPT was dissolved in 10.0 mL of PBS and then transferred into a dialysis bag (MWCO = 3500 Da). After that, the dialysis bag was put into 50.0 mL of release medium at 37 °C with continuous vibration of 70 rpm. At desired time intervals, 2.0 mL of release medium was taken out, and an equal volume of fresh PBS was replenished. The amount of released HCPT was determined by UV–Vis spectrophotometry at λ = 365 nm using a standard curve method.

### 2.7. HCPT Uptake

MCF-7 cells (a human breast cancer cell line) were cultured in complete high glucose Dulbecco’s modified Eagle’s medium (HG-DMEM) supplemented with 10% (*v/v*) fetal bovine serum (FBS), penicillin (50 IU mL^−1^), and streptomycin (50 IU mL^−1^) at 37 °C in 5% (*v/v*) carbon dioxide (CO_2_). In drug uptake assay, MCF-7 cells were planted into 96-well plates for 24 h and then incubated with different HCPT formulations at an HCPT dosage of 10.0 μg·mL^−1^ for 2, 4, 6, or 10 h. After washing three times with ice-cold PBS solution, the cells were treated with lysate (1% (*v/v*) Triton X-100 + 0.1% (*w/v*) sodium dodecyl sulfate (SDS)), and then sodium hydroxide (NaOH) was added to dissolve the internalized HCPT. After these treatments, the plate was measured at 384 nm by a Bio-Rad 680 microplate reader (Hercules, CA, USA).

### 2.8. Cytotoxicity Assays

The relative cytotoxicities of blank micelles, HCPT-loaded micelles, and free HCPT were conducted toward MCF-7 cells by an MTT assay. In brief, MCF-7 cells at a density of 8000 cells per well were seeded in 96-well plates in 200.0 μL of HG-DMEM and further incubated at 37 °C for 24 h. Then, the blank micelles in 200.0 μL of HG-DMEM with concentrations from 1.6 to 100.0 μg·mL^−1^ were added into each well. After coincubation for 72 h, a standard MTT assay was used to analyze the cell viability according to the standard protocol. In addition, the cellular proliferation inhibition capabilities of various HCPT formulations at an HCPT dosage of 0.16 to 10.0 μg·mL^−1^ after 48 or 72 h incubation were also tested by an MTT assay. The cell viability was calculated from Equation (3):

(3) $$\text{Cell viability}(\%)=\frac{A_{\mathrm{sample}}}{A_{\mathrm{control}}}\times 100\%.$$

In Equation (3), *A*_sample_ and *A*_control_ represented the absorbances of sample and control wells, respectively.

## 3. Results and Discussion

### 3.1. Syntheses and Characterizations of PLA–PEG–PLA Triblock Copolymers

**Figure 1:**
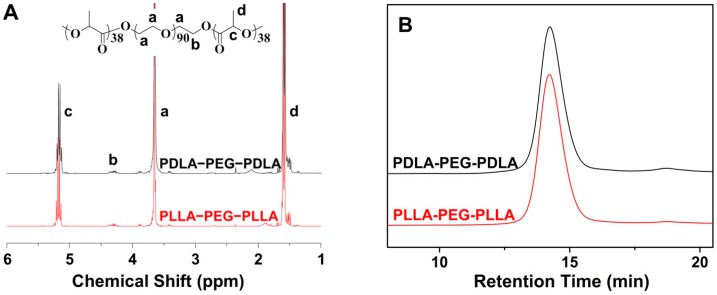
^1^H NMR spectra (in CDCl_3_) (**A**) and GPC chromatograms (**B**) of PDLA–PEG–PDLA and PLLA–PEG–PLLA copolymers.

As depicted in Scheme 1, the amphiphilic enantiomeric PLA–PEG–PLA copolymers were synthesized through the ROP of LA with PEG as a macroinitiator and Sn(Oct)_2_ as a catalyst. The structure and composition of synthesized copolymers have been determined by ^1^H-NMR and GPC. As shown in Figure 1A, there were three main characteristic peaks attributed to the protons in the copolymers: methine proton (–C*H*(CH_3_)–) and methyl proton (–C*H*_3_) at 5.2 and 1.6 ppm, respectively, which belonged to the PLA segments, respectively, while the signal of methylene proton (–C*H*_2_C*H*_2_O–) in PEG segment appeared at 3.6 ppm. The well assigned resonance peaks demonstrated the syntheses of copolymers. Furthermore, from the ratio of the peak area at 1.6 and 3.6 ppm, the degrees of polymerization (DPs) of both enantiomeric copolymers were detected to be 76 (Table 1). The precise chemical structures of copolymers provided the indispensable basis for the follow-up mechanism research of stereocomplex interaction. According to the DP of copolymers, the *M*_n_ was calculated to be 9500 g·mol^−1^ and listed in Table 1. In addition, the molecular weight distributions (polydispersity indices, PDIs = *M*_w_/*M*_n_) were determined to be 1.14 and 1.13 for PDLA–PEG–PDLA and PLLA–PEG–PLLA copolymers, respectively, by GPC (Figure 1B), indicating the nearly monodisperse molecular weights. As revealed in Table 1, It should be noted that the *M*_n_ measured by GPC was relatively higher than that from ^1^H NMR due to the structural difference between the resultant copolymers and monodisperse polystyrene standards, which were used to generate the calibration curve in GPC assessments [19].

### 3.2. Self-Assembly Behaviors and Mechanism of Stereocomplex Interaction

It is well-known that the amphiphilic copolymers can self-assemble into nano-objects in a selective solvent induced by the microphase separation, which have been widely applied to fabricate drug delivery systems [21,22]. By the same principle, the amphiphilic PLA–PEG–PLA copolymers and the equimolar mixture of enantiomeric copolymers spontaneously form into micelles in aqueous solution, that is, PDM, PLM, and SCM, respectively.

**Figure 2:**
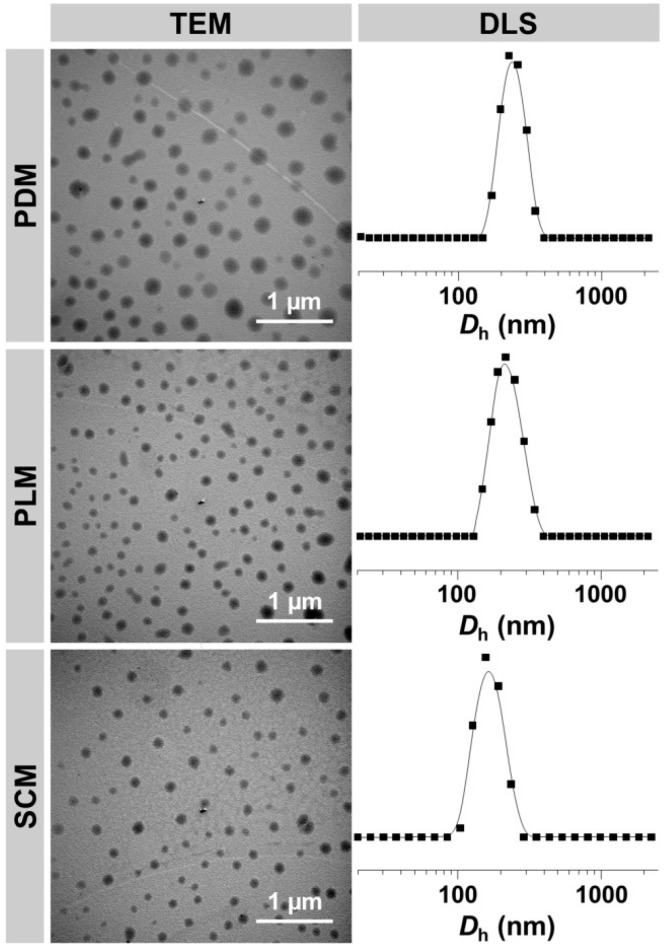
Typical TEM micrographs and *D*_h_s of PDM, PLM, and SCM.

In order to characterize the morphologies and sizes of these micelles, TEM and DLS were employed. As shown in Figure 2, the micelles of PDM, PLM, and SCM showed spherical morphologies with the respective average diameters of appropriate 200, 140, and 125 nm. In contrast, the DLS measurements revealed that the hydrodynamic diameters (*D*_h_s) of these micelles were 231 ± 4, 216 ± 5, and 164 ± 4 nm, respectively (Table 2). All the results demonstrated that SCM had the smallest size. It might be contributed to the formation of stereocomplex interaction between enantiomeric PLA–PEG–PLA copolymers, which strongly improved the compactness and stability of micelle [9,23]. The scale values of these micelles detected by DLS were bigger than those from TEM measurements, and the phenomenon should be related to the hydration states of micelles in TEM detections [24]. In addition, all of the above micelles with the diameters of around 200 nm were appropriate for passively targeting to tumor tissue through the enhanced permeability and retention (EPR) effect [25].

Furthermore, the CMCs of these micelles were investigated by a pyrene-probe-based fluorescence technique for demonstrating the formation of micelles. As listed in Table 2, the CMCs of PDM, PLM, and SCM were calculated to be 0.072, 0.061, and 0.035 mg·mL^−1^, respectively, by the plots of fluorescence intensity ratios of *I*_338_/*I*_336_ *vs.* logarithmic concentrations (lg*c*s) of these copolymers. Compared with PDM and PLM, SCM showed the lowest CMC because of the formation of tightly packed isotactic hydrophobic cores and the stereocomplex interaction between them.

**Figure 3:**
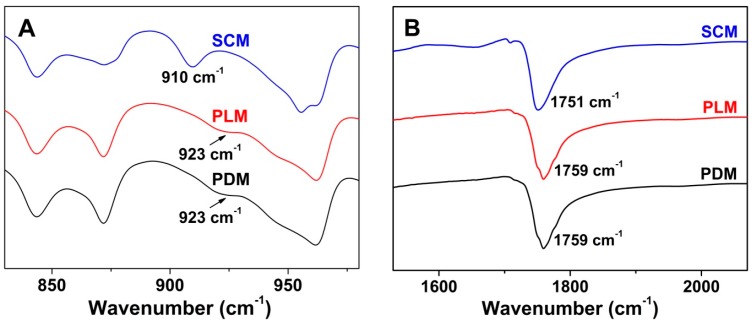
Crystallization peak variations of PDM, PLM, and SCM at ranges of 830–980 cm^−1^ (**A**) and 1530–2070 cm^−1^ (**B**).

**Figure 4:**
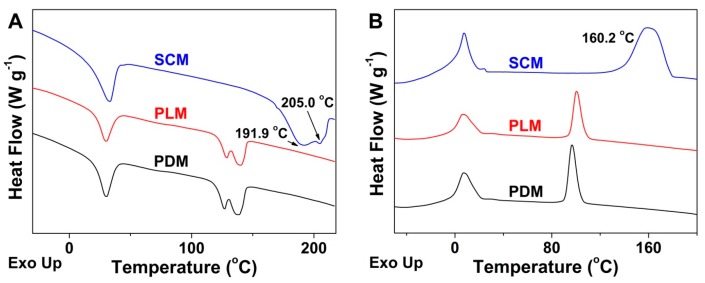
DSC thermal graphs of PDM, PLM, and SCM during heating (**A**) and cooling processes (**B**).

**Figure 5:**
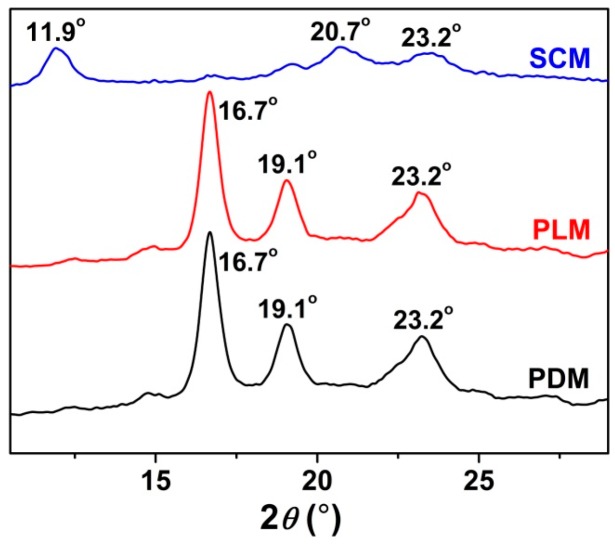
WAXD profiles of PDM, PLM, and SCM.

To further confirm the successful formation and reveal the mechanism of stereocomplex interaction in SCM, the assessments of FT-IR spectra, DSC, and WAXD were performed to reveal the characteristic thermal and microcrystalline properties of freeze-dried micelles. As shown in Figure 3, the appearance of signal at 910 cm^−1^ attributed to the PLA stereocomplex microcrystal, the disappearance of PLA crystallization peak at 923 cm^−1^, and the blue shift of carbonyl absorption (ν_C=O_) from 1759 to 1751 cm^−1^ in the SCM spectrum demonstrated the formation of stereocomplex interaction in the core of SCM [5]. In the DSC curves of Figure 4, the upregulated melting points at 191.9 and 205.0 °C, and the emerging crystallization peak at 160.2 °C of stereocomplex PLA in the core of SCM revealed the successful formation of PLA stereocomplex microcrystals [26]. Moreover, the typical WAXD patterns of PDM, PLM, and SCM were illustrated in Figure 5. The diffraction peaks of PDLA and PLLA microcrystals appeared at 2θ = 16.7° and 19.1°, while those of stereocomplex microcrystals from two equimolar enantiomeric copolymers were at 2θ = 11.9° and 20.7°, which adequately described the stereocomplex formation overall crystallization process. In addition, the PEG microcrystals in PDM, PLM, and SCM exhibited the same diffraction peak at 2θ = 23.2°. All the above results indicated that the PLA stereocomplex interaction and microcrystals were formed in the core of SCM.

### 3.3. In Vitro HCPT Loading and Release

To further reveal the superiority of SCM in the field of controlled drug delivery, HCPT, a clinical antineoplastic agent with a very low solubility in water, was loaded into the hydrophobic cores of these micelles. As shown in Table 2, the DLCs of PDM/HCPT, PLM/HCPT, and SCM/HCPT were 10.16, 11.93, and 12.96 wt %, while the DLEs of them were 75.41, 90.29, and 99.22 wt %, respectively. Compared with PDM/HCPT and PLM/HCPT, SCM/HCPT exhibited the highest DLC and DLE. It should be attributed to the formation of stable stereocomplex microcrystals of enantiomeric PLA in micellar cores [17]. Moreover, the HCPT-loaded micelles exhibited higher scales compared with the blank ones, owing to the HCPT-filled micellar cores.

**Figure 6:**
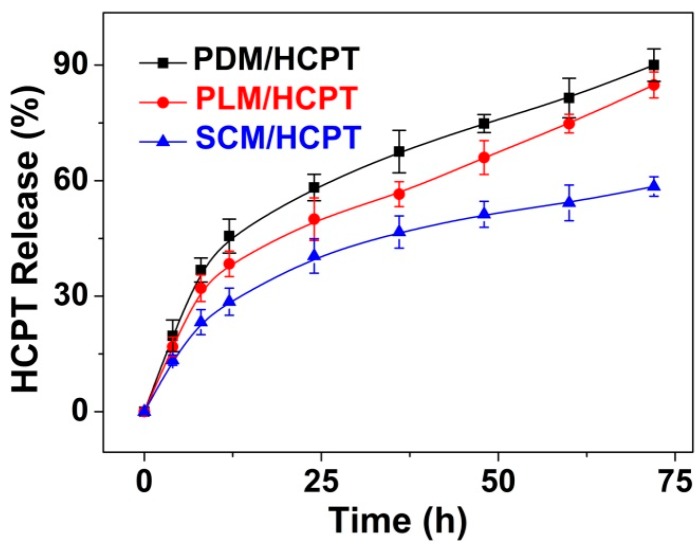
Release behaviors of PDM/HCPT, PLM/HCPT, and SCM/HCPT in PBS at pH 7.4, 37 °C. Each set of data is presented as mean ± standard deviation (SD; *n* = 3).

The *in vitro* release behaviors of these micelles were investigated in PBS at pH 7.4, 37 °C. The cumulative release percentages of HCPT-loaded micelles *vs.* time were shown in Figure 6. It was revealed that no burst release was observed in all these HCPT-loaded micelles. SCM/HCPT exhibited a much slower release rate compared to PDM/HCPT and PLM/HCPT due to the interactions between stereocomplex microcrystals and drugs, and slow degradation of stereocomplex PLA core, which also indicated the enhanced stability of SCM [27].

### 3.4. Upregulated Cellular Uptake and Antitumor Activity of SCM/HCPT

**Figure 7:**
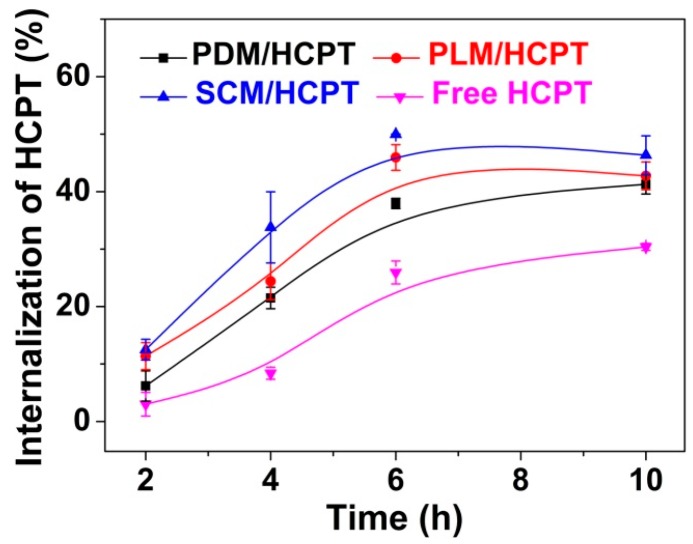
Cellular uptake of HCPT toward MCF-7 cells after incubation with PDM/HCPT, PLM/HCPT, SCM/HCPT, or free HCPT for various durations. Data are shown as mean ± SD (*n* = 4).

The cellular uptakes of HCPT-loaded micelles and free HCPT toward MCF-7 cells were demonstrated by UV–Vis spectrophotometry described by Ma *et al.* [28]. As shown in Figure 7, SCM/HCPT exhibited the highest cellular uptake of HCPT compared to the other two HCPT-loaded single component micelles and free HCPT throughout the test duration of 10 h because of the minimum extracellular release and efficient cell internalization. In detail, about 50% of HCPT was internalized by MCF-7 cells in the SCM/HCPT group, while appropriate 45%, 40%, and 30% of HCPT were uptaken in the PDM/HCPT, PLM/HCPT, and free HCPT groups, respectively, after incubation from 6 to 10 h. The results indicated the advantage of SCM as a vehicle for the effective intracellular uptake of drug.

**Figure 8:**
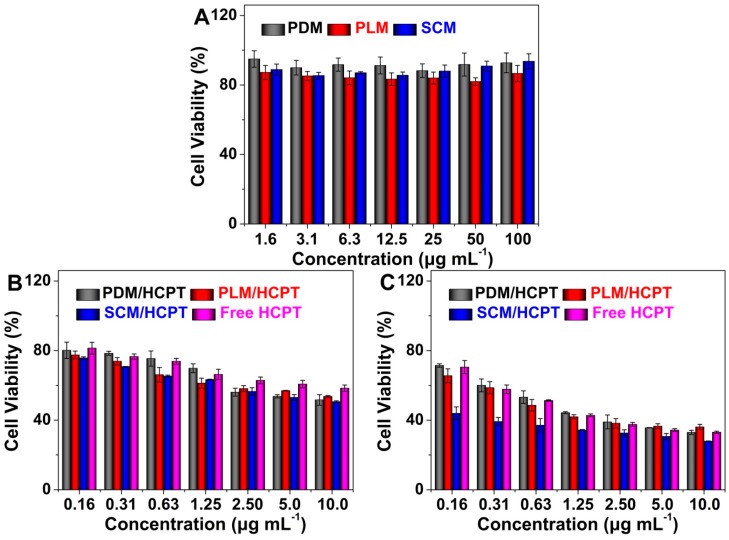
Relative viabilities of MCF-7 cells after incubation with blank micelles for 72 h (**A**); or HCPT-loaded micelles or free HCPT for 48 h (**B**) or 72 h (**C**). Data are shown as mean ± SD (*n* = 4).

Furthermore, the cytotoxicities of blank micelles, the HCPT-loaded micelles, and free HCPT were investigated against MCF-7 cells by an MTT assay. As depicted in Figure 8A, blank micelles did not show obvious toxicities toward MCF-7 cells even at a high concentration up to 100.0 µg·mL^−1^ after coculture for 72 h, indicating their excellent biocompatibility. The proliferation inhibition capabilities of HCPT-loaded micelles and free HCPT were shown in Figure 8B,C. After coincubation for 48 h, PLM/HCPT and SCM/HCPT exhibited comparable cytotoxicities, which were higher than PDM/HCPT and free HCPT (Figure 8B). More fascinatingly, SCM/HCPT exhibited the most effective proliferation inhibition efficacy on MCF-7 cells compared to those of the HCPT-loaded micelles with single components and free HCPT after coincubation for 72 h (Figure 8C). The results might be attributed to greater stability of SCM/HCPT, which guaranteed a larger amount of HCPT to be internalized into MCF-7 cells by endocytosis and more persistent HCPT to be released in tumor cells [9]. Furthermore, free HCPT exhibited the worst antitumor efficacy due to the lowest efficiency of cell internalization. The half maximal inhibitory concentrations (IC_50_s) of PDM/HCPT, PLM/HCPT, SCM/HCPT, and free HCPT were calculated to be 1.04 ± 0.22, 0.84 ± 0.28, 0.03 ± 0.03, and 0.89 ± 0.16 µg·mL^−1^ after incubation for 72 h, respectively. The SCM/HCPT was demonstrated to exhibit the lowest IC_50_, which further proved the most effective antineoplastic efficacy of SCM/HCPT quantitatively.

## 4. Conclusions

In summary, two enantiomeric PLA–PEG–PLA copolymers with regular and exact chemical structures were synthesized. The obtained amphiphilic copolymers and their equimolar mixture self-assembled into micelles in aqueous solution driven by microphase separation. The stereocomplex interaction and microcrystals were demonstrated to be formed in the core of SCM, which determined the smallest diameter and CMC. HCPT was loaded into SCM with the highest DLE of near 100 wt %. In addition, SCM/HCPT exhibited decreased drug release, enhanced intracellular uptake, and upregulated proliferation inhibition efficacy in comparison to both PDM/HCPT and PLM/HCPT. Therefore, the stereocomplex-reinforced PLA-containing micelles hold great promise as stable and satisfactory drug delivery systems for controlled drug delivery.

## Figures and Tables

**Table 1:** Characterization of PLA–PEG–PLA copolymers.

Polymer	DP of lactic acid ^1^		*M*_n_ (g·mol^−1^) ^2^	*M*_n_ (g·mol^−1^) ^3^	PDI ^3^
	Theoretical DP	Actual DP ^2^			
PDLA–PEG–PDLA	80	76	9,500	17,460	1.14
PLLA–PEG–PLLA	80	76	9,500	17,550	1.13

^1^ A monomer molecule of LA forms into two structural units of lactic acid after polymerization; ^2^ Determined by ^1^H-NMR in CDCl_3_; ^3^ Determined by GPC with THF as an elution.

**Table 2:** Properties of PDM, PLM, and SCM without and with HCPT.

Micelle	CMC (mg·mL^−1^)	*D*_h_ (nm)		DLC (wt %)	DLE (wt %)
		Blank	Load		
PDM	0.072	231 ± 4	286 ± 6	10.16	75.41
PLM	0.061	216 ± 5	274 ± 5	11.93	90.29
SCM	0.035	164 ± 4	270 ± 5	12.96	99.22
